# LPS-Induced Systemic Inflammation Affects the Dynamic Interactions of Astrocytes and Microglia with the Vasculature of the Mouse Brain Cortex

**DOI:** 10.3390/cells12101418

**Published:** 2023-05-17

**Authors:** Evangelia Xingi, Paraskevi N. Koutsoudaki, Irini Thanou, Minh-Son Phan, Maria Margariti, Anja Scheller, Jean-Yves Tinevez, Frank Kirchhoff, Dimitra Thomaidou

**Affiliations:** 1 Light Microscopy Unit, Hellenic Pasteur Institute, 11521 Athens, Greece; 2 Neural Stem Cells and Neuroimaging Group, Department of Neurobiology, Hellenic Pasteur Institute, 11521 Athens, Greece; 3 Institut Pasteur, Université de Paris, Image Analysis Hub, F-75015 Paris, France; 4 Molecular Physiology, Center for Integrative Physiology and Molecular Medicine (CIPMM), University of Saarland, 66421 Homburg, Germany

**Keywords:** astrocytes, microglia, 2-photon imaging, NVU, LPS, blood vessels, AQP4

## Abstract

The Neurovascular Unit (NVU), composed of glia (astrocytes, oligodendrocytes, microglia), neurons, pericytes and endothelial cells, is a dynamic interface ensuring the physiological functioning of the central nervous system (CNS), which gets affected and contributes to the pathology of several neurodegenerative diseases. Neuroinflammation is a common feature of neurodegenerative diseases and is primarily related to the activation state of perivascular microglia and astrocytes, which constitute two of its major cellular components. Our studies focus on monitoring in real time the morphological changes of perivascular astrocytes and microglia, as well as their dynamic interactions with the brain vasculature, under physiological conditions and following systemic neuroinflammation triggering both microgliosis and astrogliosis. To this end, we performed 2-photon laser scanning microscopy (2P-LSM) for intravital imaging of the cortex of transgenic mice visualizing the dynamics of microglia and astroglia following neuroinflammation induced by systemic administration of the endotoxin lipopolysaccharide (LPS). Our results indicate that following neuroinflammation the endfeet of activated perivascular astrocytes lose their close proximity and physiological cross-talk with vasculature, an event that most possibly contributes to a loss of blood–brain barrier (BBB) integrity. At the same time, microglial cells become activated and exhibit a higher extent of physical contact with the blood vessels. These dynamic responses of perivascular astrocytes and microglia are peaking at 4 days following LPS administration; however, they still persist at a lower level at 8 days after LPS injection, revealing incomplete reversal of inflammation affecting the glial properties and interactions within the NVU.

## 1. Introduction

Neuroinflammation plays an important role in the progression of a variety of chronic neurological/neurodegenerative diseases. A common inflammatory feature of neurodegenerative diseases is the activation of microglia and astrocytes, which constitute the two major cell populations comprising the brain parenchyma compartment of the Neurovascular Unit (NVU) [1,2]. Astrocytes play a major role in CNS blood flow regulation and the exchange of nutrients and other functional signals between the peripheral blood and brain parenchyma. This regulation is achieved by extension of their specialized processes called endfeet to the blood vessel walls. Astrocytic endfeet that enwrap the vasculature are in physical contact with the basal lamina component of the vessel wall, forming together with perivascular pericytes and neurons a dynamic interface characterized as the Neurovascular Unit (NVU) [3]. Under physiological conditions, perivascular astrocyte endfeet express molecules that maintain the integrity of the blood–brain barrier (BBB), including enzymatic and transporter proteins, the most important one being the water channel Aquaporin 4 (AQP4) [4]. A number of CNS diseases, such as multiple sclerosis [5], major depressive disorder [6] and ischemia [7], are marked by retraction or separation of astrocytic endfeet from blood vessels, a phenotype often accompanied by vascular deficits indicative of the BBB breakdown, such as altered BBB permeability or elevated CSF-to-serum albumin ratio. Microglial cells are another important partner of the NVU, also implicated in BBB integrity and homeostasis, continuously surveying their surrounding environment and rapidly repairing the BBB following acute vessel injury [8].

Physical interactions between microglia and astrocytes under physiological conditions are substantial as structural components of the NVU. Microglia promote astrocyte differentiation [9] while astrocytes regulate microglial phenotypes [10], indicating a bidirectional regulation between the glial cell populations that contributes to vascular development and BBB integrity. At the same time, cross-talk between microglia and astrocytes occurs through a variety of molecule signals, such as adenosine triphosphate (ATP), cytokines and chemokines [11].

Following systemic inflammation, microglia and astrocytes revert to a proinflammatory state, called M1 and A1, respectively, which is to a large extent regulated by the reciprocal interaction of the two cell populations through the expression of proinflammatory cytokines and altered composition of gliotransmitters secreted [1,12]. More specifically it has been shown that A1 reactive astrocytes are induced by activated M1 microglia through secretion of cytokines Il-1α, TNFα and C1q [13], while reactive astrocytes secrete factors that promote changes in the permeability of the BBB and chemokines that modulate microglial activation and motility [14,15].

Despite the wealth of data linking NVU function to microglia–astrocytes cross-talk and inflammatory responses during the progression of CNS diseases, there are a few data monitoring the dynamic physical interactions of the two glial cell populations with brain vasculature following systemic inflammation and how these may be interconnected. These dynamic interactions can be captured by in vivo imaging, which allows the study of cellular responses to neuroinflammation in the living brain in real time [16,17]. To this end, we used 2-photon laser scanning microscopy (2P-LSM) intravital imaging of the cortex of transgenic mice, to visualize and quantify the dynamic interactions of microglia and astroglia with blood vessels after neuroinflammation evoked by systemic administration of the bacterial wall endotoxin lipopolysaccharide (LPS), which we combined with immunofluorescence analysis of the expression levels and localization of AQP4 during inflammatory response progression. LPS is considered a potent activator of neuroinflammation after its peripheral administration and has been extensively used in AD, PD, ALS and HD animal models [18]. Our data indicate that following LPS administration the endfeet of activated perivascular astrocytes lose their proximity to the vasculature while activated microglial cells exhibit an opposite response pattern, as the extent of contact between their processes/cell bodies and blood vessels increases. These dynamic responses of perivascular astrocytes and microglia are peaking 4 days following LPS administration; however, they persist at a lower level at the longer time point of 8 days. The prolonged retraction of astrocytic endfeet is possibly linked to the sustained very low levels of AQP4, implying that AQP4 may be implicated in the regulation of endfeet kinetics during systemic inflammation.

## 2. Materials and Methods

### 2.1. Experimental Animals

To visualize the morphology of microglia with 2-photon microscopy, we used heterozygous Cx3cr1-EGFP transgenic mice of both sexes expressing enhanced green fluorescent protein (EGFP) knocked into the Cx3cr1 locus, which is specific for microglia, macrophages and monocytes [19] in C57BL/6J background. To visualize the morphology of astrocytes, we used hGFAP-ECFP transgenic mice expressing enhanced cyan fluorescent protein (ECFP) under the control of the human GFAP promoter which is specific for astrocytes in an FVB/N genetic background [20]. To a priori estimate the number of mice needed for intravital imaging experiments, we used the G*Power software, setting as effect size the microglial/astroglia cell body volume, and determined that a sample size of 5 animals per experimental group was needed to extract statistically significant results. Following this estimation and adhering to the principles of the 3 + 1Rs and ARRIVE, 5 Cx3cr1-EGFP and 5 hGFAP-ECFP mice were used for imaging baseline condition and all time points p.i. of LPS. For confocal microscopy experiments, we used 9- to 11-week-old wild-type C57BL/6J mice, both male and female, *n* = 3–4 for each condition.

### 2.2. Cranial Window Surgery

Cranial window surgery was performed in 6- to 8-week-old Cx3cr1-EGFP or hGFAP-ECFP transgenic mice. Following anesthesia with ketamine (100 mg/kg), xylazine (15 mg/kg) and acepromazine (2.5 mg/kg), the skull was exposed and cleaned. A circular (~3 mm in diameter) craniotomy was performed with a driller over the somatosensory cortex. The bone was gently lifted, ACSF (Tocris Bioscience, Bristol, UK) was applied to clean and remove dural bleedings and a glass window (3 mm diameter) was placed over the brain surface. A custom-made head bar was positioned over the craniotomy and was firmly affixed to the skull with cyanoacrylate glue and dental cement (Unifast Trad, Accord Corporation, Bangkok, Thailand). The edges of the cranial window were also sealed with dental cement. Imaging sessions of living animals started 3 weeks after cranial window surgery (when mice were aged 9- to 11-week old), to allow inflammation resulting from the surgical procedure to resolve.

### 2.3. 2-Photon Imaging

For each imaging time point, Cx3cr1-EGFP or hGFAP-ECFP transgenic mice were anesthetized as described in the previous paragraph and the vascular marker Rhodamine B dextran (M.W. 70 KDa, Sigma-Aldrich, St. Louis, MO, USA) was intravenously injected (3%, 50 μL). Their temperature was monitored and kept at 37 °C using a heating pad during the entire imaging sessions and until their recovery from anesthesia. Animals were head restrained at the stage of a Leica TCS-SP5 2-photon microscope (Leica Microsystems GmbH, Mannheim, Germany) equipped with a 25× water-injection objective (NA 0.9, Leica Microsystems GmbH, Mannheim, Germany) and a Ti-sapphire laser Mai Tai DeepSee (Spectra Physics, Milpitas, CA, USA) tuned to an excitation wavelength of 900 nm. Imaging was performed 100 μm below the dura in the somatosensory cortex. Image z-stacks were typically 1024 × 1024 pixels with a voxel size of 0.144 (x) × 0.144 (y) × 0.988 (z) μm acquired for all samples. A 560 nm dichroic mirror was used to separate 525/50 nm (green channel for EGFP fluorescence detection) or 483/32 nm (cyan channel for ECFP fluorescence detection) and 585/40 nm (red channel for Rhodamine B dextran fluorescence detection) emission filters and fluorescence was collected using NDD detectors.

### 2.4. Systemic LPS Administration

LPS (Lipopolysaccharides from *Escherichia coli* serotype 055:B5; Sigma-Aldrich, St. Louis, MO, USA) was administered to induce systemic inflammation. For 2-photon in vivo imaging experiments of mouse brains, 9- to 11-week-old Cx3cr1-EGFP (*n* = 5) or hGFAP-ECFP (*n* = 5) transgenic animals with cranial window firmly attached to the skull were treated with LPS with the following scheme: Untreated mice had a baseline 2-photon imaging session through their cranial window and two doses of LPS (5 mg/kg, i.p.) were administered with a time interval of 24 h (day −1 and day 0) starting the next day after baseline imaging. Two more 2-photon imaging sessions were performed for each animal at day 4 and day 8 p.i. of the second LPS dose. For confocal imaging experiments, adult C57BL/6J mice (9- to 11-week old) were injected with the same scheme of LPS as previously and sacrificed at 1 (LPS 1 day, *n* = 3), 4 (LPS 4 days, *n* = 4) and 8 days (LPS 8 days, *n* = 4) after the second LPS injection (p.i.). A group of untreated animals of the same age was used as a control (*n* = 4).

### 2.5. Immunohistochemistry

Mice were deeply anesthetized by isoflurane inhalation and perfused with 4% paraformaldehyde (PFA) via the left cardiac ventricle. Brains were removed, post-fixed in 4% PFA overnight and then cryoprotected in 20% sucrose overnight. After cryoprotection brains were frozen in OCT compound and cut into 20 μm thick coronal sections on a cryostat (Leica CM1900, Nussloch, Germany), collected on silane-coated slides and stored at −20 °C until further processing.

For immunofluorescence experiments, sections were left for 15 min at room temperature, washed in PBS and blocked with 5% normal donkey serum (Merck-Millipore, Burlington, MA, USA) in PBT (0.5% Triton X-100/PBS) for 1 h. Incubation with primary antibodies was performed overnight at 4 °C. After PBS wash, sections were subsequently incubated with secondary antibodies in PBS at room temperature (RT) for 3 h. Finally, sections were washed and embedded with Mowiol (Calbiochem, San Diego, CA, USA).

The following antibodies were used for staining: for astrocytes rabbit anti-GFAP (1:500, Z0334, Agilent Technologies, Santa Clara, CA, USA), or mouse anti-GFAP (1:200, G-3893, Sigma-Aldrich, St. Louis, MO, USA), for vessels goat anti-CD31 (1:200, AF3628, R&D Systems, Minneapolis, MN, USA), for astrocyte endfeet mouse anti-AQP4 (1:200, sc-32739, Santa Cruz Biotechnology, Dallas, TX, USA) and for microglia rabbit anti-Iba1 (1:500, Wako Chemicals, Osaka, Japan 019-19741). Secondary antibodies used were conjugated with AlexaFluor 488 (green), 546 (red), 647 (1:600, all from Biotium, Fremont, CA, USA) and Hoechst (1:600) for nuclei staining.

Images were acquired using a Leica TCS-SP5 confocal microscope (Leica Microsystems GmbH, Mannheim, Germany) with a 40× objective (NA 1.3) using a pixel size of 0.361 × 0.361 × 1 μm and a Leica TCS-SP8 confocal microscope (Leica Microsystems GmbH, Mannheim, Germany) with a 40× objective (NA 1.3) using a pixel size of 0.284 × 0.284 × 1 μm. Images were typically 1024 × 1024 pixels.

### 2.6. Image Analysis

Image analysis was performed with the Fiji software [21], Python Jupyter notebooks [22] and Imaris (v 9.3.1, Bitplane, Oxford Instruments-Andor, Belfast, UK). We provide the Fiji macros and notebooks for the entire image analysis pipeline above and they can be downloaded from the public code hosting website: https://gitlab.pasteur.fr/iah-public/ptrmiad-thomaidou (accessed on 29 March 2023).

To measure cell body volumes of microglia and astrocytes in 2-photon images, we first applied auto-thresholding using Renyi Entropy [23] to segment the cell body. Morphological opening with radii = 5.5 and 2 pixels in x, y and z was then used to exclude the remaining processes from the segmented objects. The touching cell bodies were split using watershed separation in the MorphoLibJ plugin [24]. This tool was used to remove spurious detection by setting a minimum volume of 200 µm^3^, a minimum mean intensity of 60 and a maximum flatness of 3.5 for objects.

To measure the contact ratio between microglia, astrocytes and the blood vessels in 2-photon images, we first smoothed the images with a Gaussian filter with sigma = 2 pixels and enhanced the image contrast by saturating pixels with a percentage of 0.7%. The intensity in the blood vessels channel was subtracted from the intensity in the microglia/astrocytes channel to remove the wavelength mixing effect. Microglia, astrocytes and the blood vessels were then segmented by auto-thresholding using the Otsu method [25], and overlapping regions were identified from their masks across z slices, from which we deduced the contact ratio.

Confocal images with AQP4 contacts to the blood vessels (CD31) were analyzed using Imaris surface-surface contact area XTension. More specifically a primary surface of the CD31 staining and a secondary of the AQP4 staining were created, and the percentage of the new surface contact area relative to the total surface area of the primary surface was estimated using the mentioned XTension.

Analyses of microglia and astrocytic reactivity were performed as quantification of the areas immunostained positive for Iba1 and GFAP, respectively. Measurements were performed on maximum intensity projections of confocal z stacks of ~30 μm using automatic thresholding Default for microglia and MaxEntropy for astrocytes in Fiji.

### 2.7. Statistics

All quantified data are presented as mean ± standard deviation (SD) values. Statistics were performed using GraphPad Prism 9.5.1. Values from all quantifications performed were initially tested for normality using the Shapiro–Wilk test. All data passed successfully the normality test. Data from 2-photon intravital imaging were further analyzed using Repeated Measures one-way ANOVA and data from confocal experiments were analyzed using Ordinary one-way ANOVA. When interactions were detected, group comparisons were performed using *t*-test assuming unequal variances for confocal data, and Tukey’s multiple comparisons test was used for 2-photon imaging data. The level of statistical significance was set at 0.05.

## 3. Results

### 3.1. LPS Systemic Administration Activates Perivascular Astrocytes and Microglia

**Figure 1:**
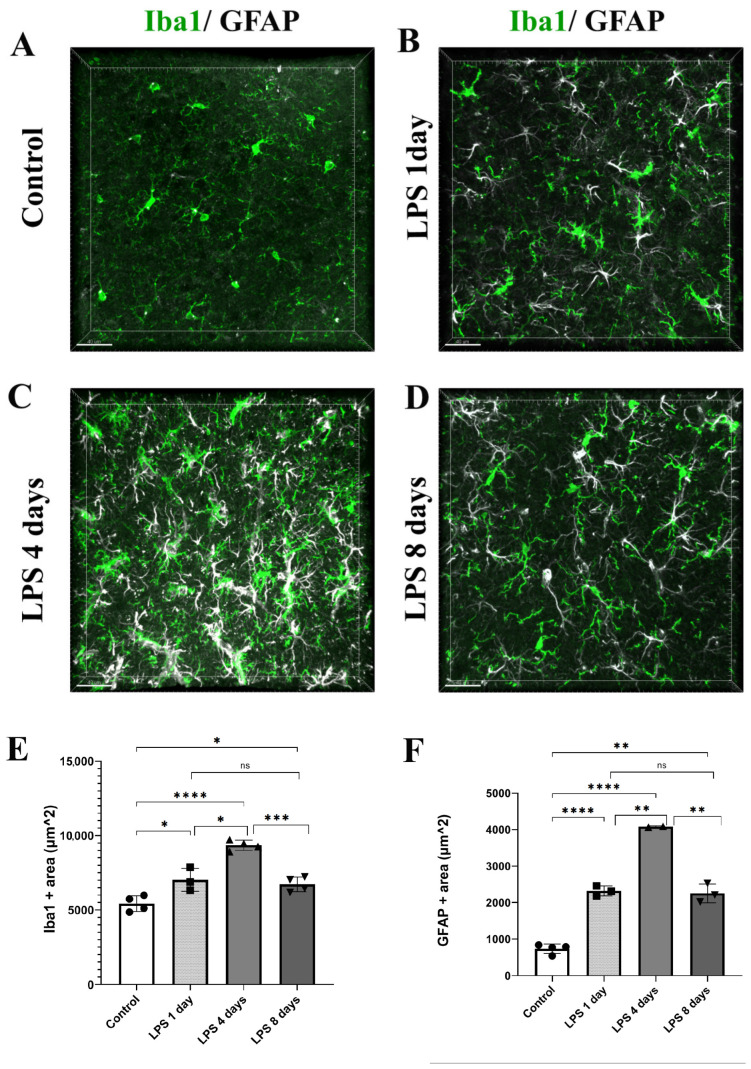
Time course of astrocytes and microglia activation following LPS-induced inflammation. (**A**–**D**) Immunofluorescence staining for microglia (Iba1, green) and astrocytes (GFAP, white) in the cortex of C57BL/6J mice cortical brain slices showed the highest Iba1 and GFAP expression levels at 4 days after LPS injection. Images were acquired with a Leica TCS SP5 confocal microscope using a 40× objective. (**E**,**F**) Quantification of Iba1 (**E**) and GFAP (**F**) immunopositive area (μm^2^) in maximum intensity projections in control mice (*n* = 4), and mice sacrificed 1 day (*n* = 3), 4 days (*n* = 4 for Iba1 and *n* = 2 for GFAP staining) and 8 days p.i. of LPS (*n* = 4). Numbers of mice and mean value of each mouse are annotated on the bars as black symbols (circle: control, square, LPS 1 day, triangle: LPS 4 days, inverted triangle: LPS 8 days). (**E**) Control vs. LPS 1 day * *p* = 0.0464, control vs. LPS 4 days **** *p* < 0.0001 and LPS 4 days vs. LPS 8 days *** *p* = 0.0002. (**F**) Control vs. LPS 1 day **** *p* < 0.0001, control vs. LPS 4 days **** *p* < 0.0001 and LPS 4 days vs. LPS 8 days ** *p* = 0.0062. Scale bar: 40 μm.

To determine the time frame during which the perivascular glial cell populations of microglia and astrocytes become activated in response to systemically induced inflammation, we administered two daily doses of LPS (5 mg/kg each) to 9- to 11-week-old C57BL/6J mice, and animals were sacrificed 1, 4 and 8 days following the second dose of LPS administration. Mouse brain sections were labeled using immunofluorescence for Iba1 and GFAP microglia and astrocytic markers, respectively (Figure 1A–D). Iba1 and GFAP immune-positive areas (μm^2^) in the somatosensory cortical area were measured on maximum intensity projections of cortical z stacks at all time points revealing that both microglia and astrocytes exhibited the highest expression of Iba1 and GFAP, respectively, 4 days following LPS administration (Figure 1E,F). More specifically, regarding microglia, in control mice, Iba1^+^ area was 5421 μm^2^ ± 526 μm^2^, whereas 1 day post-injection (p.i.) of LPS an increase was already evident (7025 μm^2^ ± 775 μm^2^). Four days p.i. of LPS Iba1 expression further increased (9347 μm^2^ ± 342 μm^2^), while at 8 days p.i. of LPS Iba1^+^ area decreased (6718 μm^2^ ± 488 μm^2^). Regarding astrocytes, in control mice, GFAP^+^ area was 733.8 μm^2^ ± 137 μm^2^, whereas 1 day p.i. of LPS it was 2319 μm^2^ ± 137 μm^2^. At the LPS 4 days p.i. time point, GFAP^+^ area further increased to 4081 μm^2^ ± 19 μm^2^, while it decreased to 2249 μm^2^ ± 257 μm^2^ 8 days p.i. of LPS.

**Figure 2:**
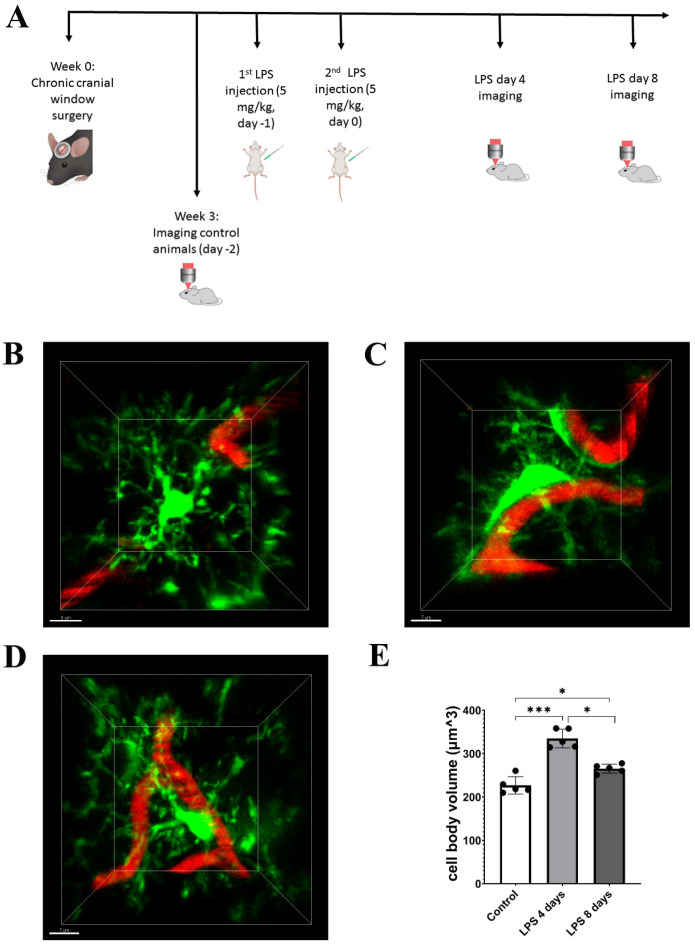
LPS-induced inflammation results in changes in microglia cell body volume and morphology. (**A**) Protocol of brain intravital 2-photon imaging following LPS systemic administration. At week 0, cranial window surgery was performed in 6- to 8-week-old Cx3xr1-EGFP or hGFAP-ECFP transgenic mice. Imaging experiments of untreated control animals were performed 3 weeks after cranial window surgery, in order to eliminate inflammation caused by the surgical procedure. For the next two sequential days, mice were injected with LPS (5 mg/kg) and imaging was performed at 4 days and 8 days after the second dose (LPS day 4 and LPS day 8 time points). (**B**–**D**) Two-photon magnified images of Cx3cr1-EGFP microglial cells (green) associated with blood vessels marked with Rhodamine B dextran (red). (**B**) An untreated control animal imaed 3 weeks after cranial window surgery. (**C**) The same animal imaged 4 days after LPS administration. (**D**) The same animal imaged 8 days after LPS administration. (**E**) The bar graph presents the quantification of the mean volume of the microglial cell soma in the three time points. Multiple images from Cx3cr1-EGFP mice (*n* = 5) were analyzed. Black circles on the bars represent mean values of each mouse measured. Control vs. LPS 4 days *** *p* = 0.0004, control vs. LPS 8 days * *p* = 0.03 and LPS 4 days vs. LPS 8 days * *p* = 0.01. Scale bar: 7 μm.

Following confocal analysis, we established a protocol of 2-photon intravital imaging of the cortex of transgenic mice expressing fluorescently labeled proteins in the microglial or astrocytic cell populations, to monitor in real time the dynamic response of microglia and astrocytes to systemically induced inflammation (Figure 2A). To this end, cortical microglia and astrocytes of Cx3cr1-EGFP and hGFAP-ECFP transgenic mice were imaged by 2-photon microscopy under baseline conditions (control). For the next 2 days (day −1 and day 0), the same mice received 5 mg/kg of LPS and were imaged at days 4 and 8 after the second dose of LPS injection. The acquired image z-stacks were analyzed using Fiji-based custom-made macros that allowed quantification of microglial and astrocytic 3D cell body volume as well as the extent of contact with blood vessels in multiple-image data sets.

The 2-photon imaging revealed the microglial cell response to LPS inflammation in greater detail, with microglia exhibiting morphological alterations indicative of an activated state, by becoming hypertrophic and acquiring a less ramified amoeboid-like elongated morphology 4 days p.i. of LPS (Figure 2B–D). More specifically, data obtained from Cx3cr1-EGFP mice showed a significant increase in the cell body volume by 47.6% (from 226.9 ± 20 μm^3^ to 334.9 ± 21.7 μm^3^) 4 days p.i. of LPS as compared to control conditions (Figure 2B,C,E). At the same time, microglia cell bodies were observed in close proximity to blood vessels (Figure 2B–D; Video S1 for LPS day 0 and Video S2 for LPS day 4). Microglia activation was reduced but persistent at 8 days p.i. of LPS, as indicated by the statistically significant lower value of microglial cell body volume (265.4 ± 10 μm^3^) in comparison to 4 days p.i. of LPS. However, microglial cell body volume at this longer time point remained increased by 16.9% as compared to the baseline condition (Figure 2B–E; Video S3). We also observed a relative restoration of ramified morphology and a lower extent of contact with blood vessels as compared to 4 days p.i. (Figure 2C,D; Video S2).

**Figure 3:**
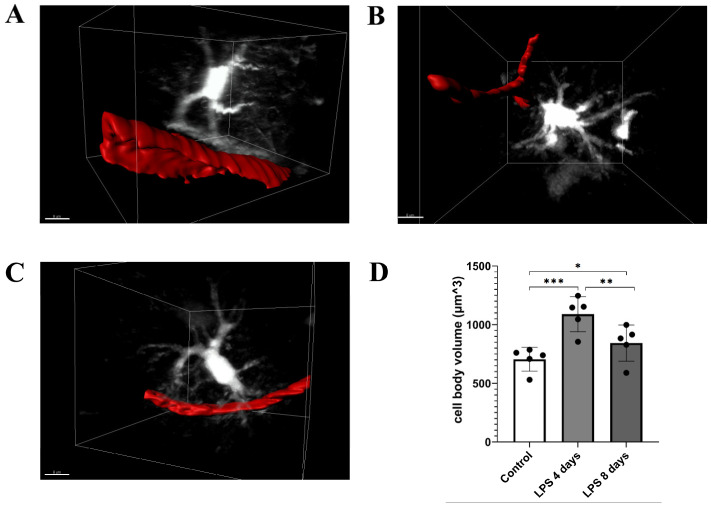
LPS-induced inflammation results in changes in astrocytes’ cell volume and morphology. 2-photon magnified images of hGFAP-ECFP astrocytes (white) associated with blood vessels marked with Rhodamine B dextran (red). For visualization purposes, blood vessels are highlighted with a surface created with Imaris. (**A**) An untreated control animal imaged 3 weeks after cranial window surgery. (**B**) The same animal imaged 4 days after LPS administration. (**C**) the same animal imaged 8 days after LPS administration. (**D**) The bar graph presents the quantification of the mean volume of the astrocytic cell soma in the three time points. Multiple images from hGFAP-ECFP mice (*n* = 5) were analyzed. Black circles on the bars represent mean values of each mouse measured. Control vs. LPS 4 days *** *p* = 0.0002, control vs. LPS 8 days * *p* = 0.029 and LPS 4 days vs. LPS 8 days ** *p* = 0.003. Scale bar: 8 μm.

LPS administration also induced a significant increase in astrocytic cell body volume 4 days p.i. indicative of astrocytic activation, which was accompanied by retraction of astrocytic endfeet from blood vessels (Figure 3A,B,D; Video S4 for LPS day 0, Video S5 for LPS day 4). Data obtained from hGFAP-ECFP transgenic mice showed a 54.5% increase in the cell body volume (from 704.8 ± 101 μm^3^ to 1089 ± 149 μm^3^). Astrocytic cell body volume decreased by 22.6% 8 days p.i. of LPS (842.8 ± 154 μm^3^), as compared to 4 days p.i. of LPS, still remaining significantly higher than in control animals (Figure 3C,D; Video S6).

### 3.2. Astrocytic Endfeet Reduce Their Close Proximity to Blood Vessels Following LPS-Induced Neuroinflammation

**Figure 4:**
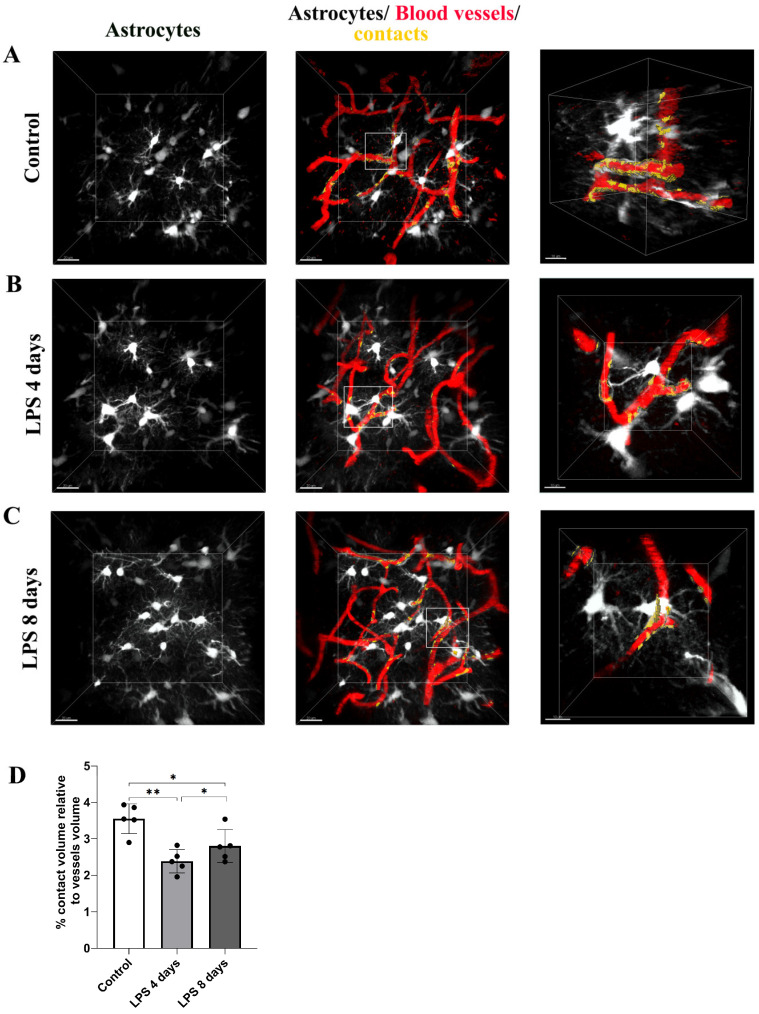
Dynamic changes in the contact of astrocytic endfeet with blood vessels following LPS-induced inflammation. 2-photon image z stacks from a single hGFAP-ECFP transgenic mouse demonstrate astrocyte retraction from cerebral vessels during systemic inflammation induced by two doses of LPS (5 mg/kg each). The left column shows astrocytes depicted in white, and the middle column shows astrocytes, blood vessels (in red) and their contacts (in yellow). The right column shows magnified images of the region indicated in the main panel (scale bar 10 μm). (**A**) An untreated control animal imaged 3 weeks after cranial window surgery. (**B**) The same animal imaged 4 days after LPS administration. (**C**) The same animal imaged 8 days after LPS administration. Scale bar: 20 μm. (**D**) The bar graph presents the quantification of the percentage of the contact volume (between astrocytes and the vessels) relative to the vessels’ volume (*n* = 5), which indicates that the contact of astrocytes to blood vessels decreases from 3.55 ± 0.41% in control to 2.38 ± 0.32% at 4 days and 2.8 ± 0.45% at 8 days p.i. of LPS. Quantification was performed using a customized Fiji macro, as described in = Section 2.6. Black circles on the bars represent mean values of each mouse measured. Control vs. LPS 4 days ** *p* = 0.0052, control vs. LPS 8 days * *p* = 0.01 and LPS 4 days vs. LPS 8 days * *p* = 0.03. Visualization of contact areas was performed using Imaris 9.3.1 software.

Analysis of 3D intravital imaging data derived from Cx3cr1-EGFP and hGFAP-ECFP mice indicated that LPS systemic administration induced dynamic changes in the contact of both perivascular microglia and astrocytes with blood vessels. To quantify in real time the extent of contact of each of the two cell populations with the cortical vasculature, we developed a Fiji-based macro to measure contact volume (of either astrocytes or microglia) to blood vessels volume in a batch mode in multiple-image data sets. Measurements of astrocytes–vessels contact volume relative to the total volume of vessels (illustrated in yellow) in hGFAP-ECFP mice (Figure 4A–C) point to a significant decrease in the contact of astrocytes to the vasculature at 4 days p.i. of LPS by 32.9% as compared to the control condition, confirming our original observation, a phenomenon which was only slightly reversed 8 days p.i. of LPS (Figure 4D).

**Figure 5:**
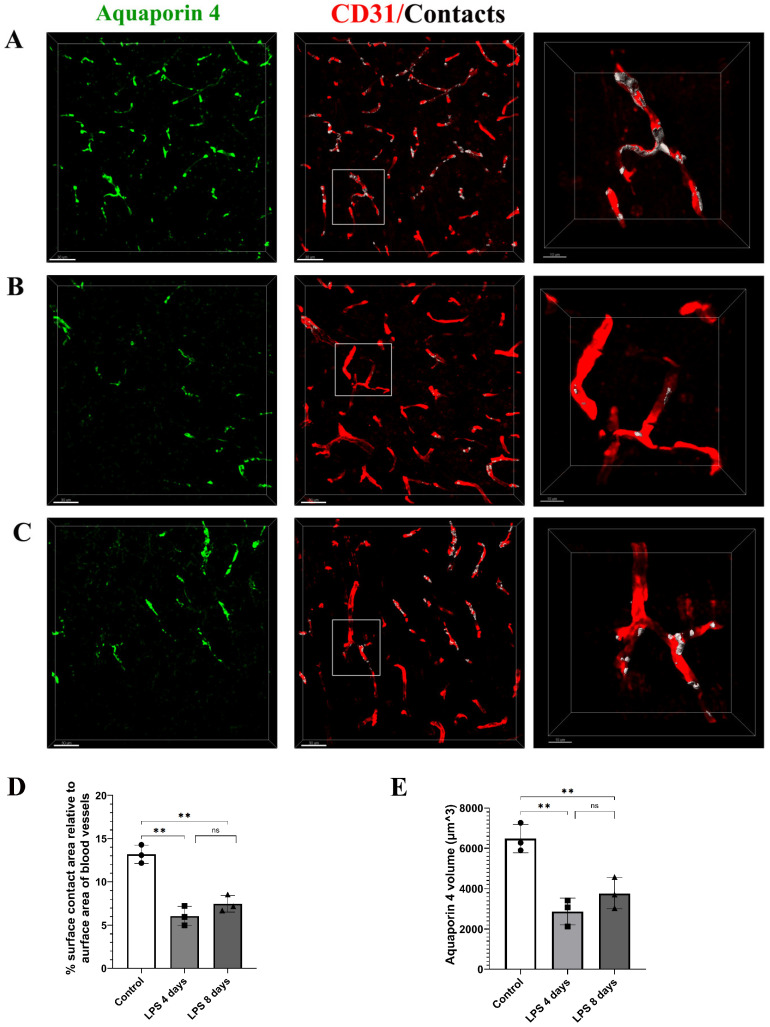
Expression of AQP4 in astrocytic endfeet following LPS-induced inflammation. Confocal images from C57BL/6J mice (9- to 11-week old) brain slices were acquired with 40× objective using a Leica TCS SP8 confocal microscope. The brain slices were immunolabeled for AQP4 (green) showing astrocytic endfeet and CD31 (red) showing blood vessels. Scale bar, 30 μm. Contact areas between the astrocytic endfeet and the blood vessels are shown in white color in the third column. The right column shows magnified images of the region indicated in the main panel (scale bar 10 μm). (**A**) Control animal which received sterile physiological saline i.p. (**B**) Mouse injected with two doses of LPS (5 mg/kg, i.p. each) for 2 consecutive days and was sacrificed at LPS day 4 time point, and (**C**) mouse injected with two doses of LPS (5 mg/kg, i.p. each) for 2 consecutive days and was sacrificed at LPS day 8 time point. (**D**) Quantitative analysis of the percentage of the surface contact area (between blood vessels and AQP4) relative to the total surface area of the blood vessels in control mice (*n* = 3) and mice sacrificed 4 (*n* = 3) and 8 days p.i. of LPS (*n* = 3). Numbers of mice and mean value of each mouse are annotated on the bars as black symbols (control: circle, LPS 4d: square, LPS 8d: invered triangle). Control vs. LPS 4 days ** *p* = 0.0014, LPS 4 days vs. LPS 8 days ns (not significant) and control vs. LPS 8 days ** *p* = 0.002. Analysis was performed using the Imaris surface-surface contact area XTension. (**E**) Quantification of AQP4 total volume (μm^3^) was measured using the Imaris v.9.3.1 surfaces module. Control vs. LPS 4 days ** *p* = 0.003, LPS 4 days vs. LPS 8 days ns (not significant) and control vs. LPS 8 days ** *p* = 0.011.

To further explore this finding, we investigated the localization pattern of the water channel AQP4, known to be localized in astrocytic endfeet under physiological conditions [26]. Immunohistochemical labeling of the cortex of C57BL/6J mice using antibodies against AQP4 and CD31 to label blood vessels revealed that in control animals the percentage of the surface contact area between AQP4 and blood vessels, relative to the surface area of blood vessels, is 13.2 ± 1.1% (Figure 5A–C). This value was severely reduced by 54% 4 days p.i. of LPS and remained low 8 days p.i. of LPS (4 days LPS: 6.06% ± 1.111%, 8 days LPS: 7.48% ± 1%) (Figure 5D). In parallel with reduced AQP4 staining in close proximity to the vasculature walls, a significant reduction in overall AQP4 expression levels was evident both at 4 and 8 days p.i. of LPS as compared to control conditions (Figure 5E). AQP4 volume in control was 6480 ± 707 μm^3^, decreasing to 2875 ± 667 μm^3^ 4 days p.i. and 3772 ± 769 μm^3^ 8 days p.i. of LPS.

### 3.3. Activated Microglia Processes Enwrap Blood Vessels Following LPS-Induced Neuroinflammation

**Figure 6:**
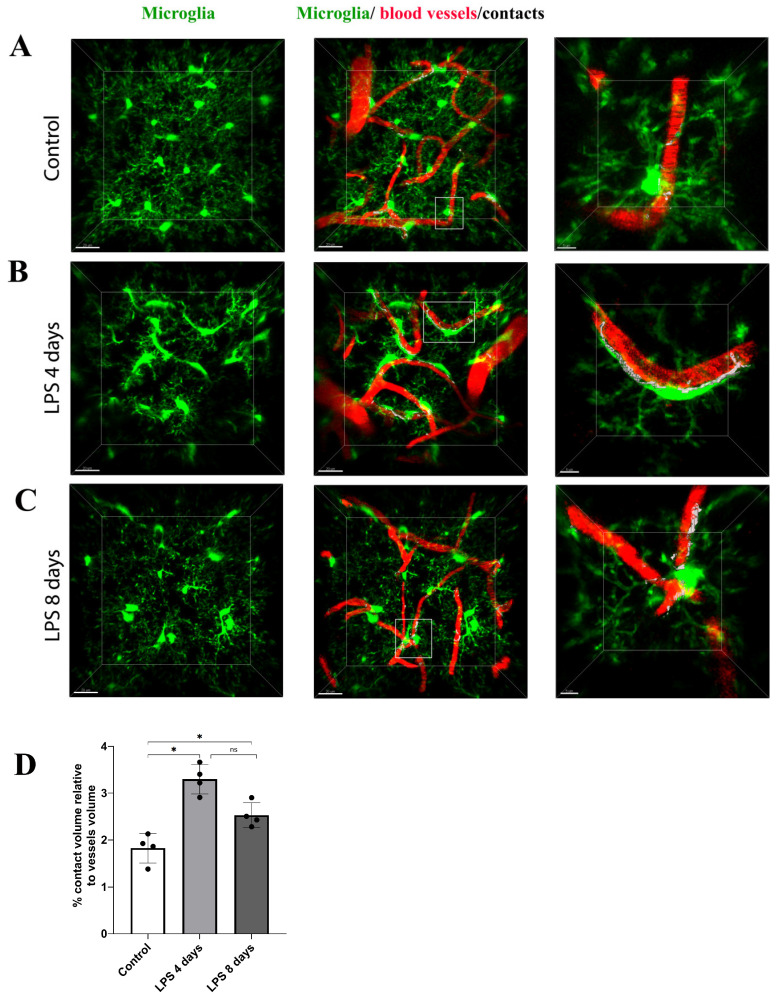
Dynamic changes in the contact of microglia processes with blood vessels following LPS-induced inflammation. 2-photon image z stacks from a single Cx3cr1-EGFP transgenic mouse demonstrate microglia migration to cerebral vessels during systemic inflammation induced by two doses of LPS (5 mg/kg each). Microglia cells are depicted in green color, blood vessels in red and their contacts in white. Close-ups of the merged images are shown in the right panels (scale bar 5 μm). (**A**) An untreated control animal imaged 3 weeks after cranial window surgery. (**B**) The same animal imaged 4 days after LPS administration. (**C**) The same animal imaged 8 days after LPS administration. Scale bar: 20 μm. Visualization was performed using Imaris software. (**D**) Bar graph presenting quantification of the percentage of the contact volume relative to the vessels volume (*n* = 4). Black circles on the bars represent mean values of each mouse measured. Control vs. LPS 4 days * *p* = 0.02, control vs. LPS 8 days * *p* = 0.049 and LPS 4 days vs. LPS 8 days ns (not significant).

To estimate the dynamic interactions of microglia with blood vessels in our model, we measured the percentage of the microglia–vessels contact volume relative to the total volume of vessels (illustrated in white) in Cx3cr1-EGFP transgenic mice (Figure 6A–C). Our data indicated that in contrast to astrocyte–vasculature interactions, activated microglial cells exhibited a significant increase in their contacts with the vasculature 4 days p.i. of LPS in comparison to the baseline condition (3.29 ± 0.31% vs. 1.82 ± 0.32%, Figure 6D), an increase that was partially reversed in LPS day 8 (2.53 ± 0.26%), however without returning to control levels.

## 4. Discussion

In this study, we used 2-photon intravital imaging to monitor and quantify in real time the activation state and dynamic physical interactions of astrocytes and microglia with the brain vasculature following brain inflammation induced by systemic administration of LPS. We report a mechanism through which both activated glial populations exhibit an opposing pattern of blood vessel coverage, with astrocytes retracting their endfeet from the vasculature at the peak of the inflammatory response at 4 days post LPS injection, while at the same time microglia processes contact and enwrap blood vessels. Through immunohistochemical stainings, we observed a reduction in AQP4 in close proximity to vessel walls, a finding confirming the removal of astrocytic endfeet, as well as a reduction in AQP4 expression overall. While most of these effects were reduced at the later time point of 8 days post LPS injection, they do not seem to be resolved, showing a transient but relatively lasting nature.

Reactivity of astro- and microglia, observed here by 2-photon imaging through changes in morphology and body size, is a well-documented effect in the frame of the neuroinflammatory response to LPS administration in various dosages [18]. In our model, as time p.i. of LPS progresses, astrocytes and microglia differentiate their kinetics of vasculature coverage, in a process that could imply a mutual regulation of the dynamic properties of spatial proximity of astrocytic endfeet and microglia processes with brain vasculature. Such astrocytes–microglia cross-talk mechanisms seem to be in place from early developmental stages, according to findings showing that microglia associate with capillary vasculature in the healthy, developing and adult brain in areas lacking astrocytic coverage [27]. Astrocytes and microglia regulation of each other’s activation has been also documented in various occasions, e.g., following TBI, with astrocyte-derived chemokine CCL7 triggering microglia activation [28]. Additionally, microglia-released IL-1β can suppress SHH expression in astrocytes inducing them to secrete proinflammatory cytokines [29] promoting BBB leakage in an in vitro BBB model. Disturbance of astrocytic A1 phenotype induction by reactive microglia either by blocking proinflammatory factors such as Il-1α, TNF and C1q [13] or by blocking or activating specific pathways in various neuroinflammatory diseases models [30,31,32] has significant neuroprotective effects. Importantly, blocking C3d+/GFAP+ A1 astrocyte phenotype induction also attenuates BBB leakage in an ischemia mouse model [33], showcasing the possible therapeutic possibilities offered by early intervention against reactive microglia-induced neurotoxic astrocytic A1 phenotype conversion in neuroinflammation. There is therefore the possibility of a proinflammatory cross-talk between astroglia and microglia during the early stages of inflammatory response in our model.

It is possible that there is a direct effect of the altered glial cell dynamics regarding their proximity to blood vessels, as observed in our study, on BBB permeability, which is often shown to be affected in the LPS-induced neuroinflammation [18,34]. Though there are many indications supporting that disruption of the association of astrocytic endfeet with brain vasculature takes place under neuroinflammatory/neurodegenerative conditions, still data concerning the extent of contribution of astrocytic endfeet coverage on BBB integrity are contradictory [35,36,37]. The role of microglia in maintaining BBB integrity during neuroinflammatory response conditions is also contradictory. Our data showing increased contact of microglial cells with blood vessels come in accordance with previous reports showing that microglial cells rapidly migrate and repair the BBB following acute vessel injury [8] or long-term LPS administration [38]. In this latter study, the involvement of microglia in BBB permeability is mediated via an endothelial cell cytokine-mediated mechanism that controls the migration of microglia to the blood vessels at initial stages of inflammation independently of its activation state, which is followed by phagocytosis of astrocytic endfeet by activated microglia, and impairment of BBB function at later stages [38]. Microglia, thus, demonstrate a dual role in BBB permeability, and while its initial migration to the vessels seems protective of BBB integrity, in later stages of the inflammatory response, it shows a detrimental effect. It is possible that in our neuroinflammation paradigm, reactive phagocytosing microglial cells participate to some extent in the observed astrocytic endfeet retraction. Whether, though, astrocytic process retraction from the vessel walls begins independently of microglia reactivity warrants further investigation. It is also possible that at the initial stages of neuroinflammation, regulation of microglial dynamic properties of proximity to blood vessels takes place in an orchestrated manner with the regulation of astroglia endfeet enwrapment to ensure more timely BBB protection.

Our data also revealed that the retraction of astrocytic endfeet from blood vessels in response to systemic inflammation is accompanied by a sharp decline of AQP4 expression and by minimal AQP4 localization at the level of blood vessel walls. A role for the AQP4 channel in the development and regulation of neuroinflammation has been shown in several animal models of neurological conditions [39,40,41], being sometimes linked to the water clearance properties of the molecule. AQP4 dysregulation could induce or exacerbate neuroinflammation through disrupted clearance of interstitial solutes that can be toxic upon accumulation or aggregation, such as Aβ or tau, as a result of altered water clearance from the brain parenchyma [39,42]. Additionally, AQP4 deficiency has been implicated in reduced cytokine release [39], while BBB disruption and subsequent proinflammatory cytokine leakage to the brain parenchyma have also been proposed as a pathway through which AQP4 dysregulation could extend proinflammatory functions [41]. Under this light, continued dysregulation of AQP4 in our model could account for the relatively persistent inflammatory response observed at 8 days p.i. of LPS. Moreover, the involvement of AQP4 in astrocytic processes plasticity [42] gives rise to the question of whether AQP4 plays a role in the extent of vessel coverage by astrocytic endfeet [43] irrespectively of its proinflammatory activity. It is possible that the severe reduction in AQP4 expression found in our experiments triggers the long-term retraction of astrocytic endfeet, highlighting its potential significant role in safeguarding BBB integrity.

Astrocytic AQP4 reduction following LPS-induced inflammation may also be one of the factors responsible—albeit in an indirect way—for the observed enwrapment of blood vessels by microglial processes [27]. Decreased levels of AQP4 have been correlated with increased microgliosis following traumatic brain injury [44], supporting that AQP4 levels and presence in close proximity to blood vessel walls may act as a cue controlling the physical contact of activated microglia with blood vessels during the inflammatory response. In support of this assumption, reports on animal models of Parkinson’s disease [39] and autoimmune encephalomyelitis [45] argue that AQP4 is a modulator of neuroinflammation via regulating the release of inflammatory cytokines such as IL1b, IL6, TNFa and ATP from astrocytes which lead to activation of microglia.

Collectively our findings indicate that astrocytes and microglia act synergistically to safeguard blood vessel coverage early upon systemic neuroinflammation. These data come in accordance with findings supporting the independent role of the astrocytes [43] and microglia [18] as mediators of vascular integrity, but they also corroborate the existence of a compensatory regulatory mechanism impacting NVU function and brain homeostasis in early stages of neuroinflammatory response. Uncovering the dynamic cross-talk of astrocytes with microglia through their physical interaction with blood vessels to maintain BBB integrity supplements the documented joint regulatory activity of the two cell populations to adjust the neuroinflammatory response to systemic inflammation through secretion of proinflammatory cytokines [13]. Our data also support a possible role of AQP4 as a linking molecule participating in the control of BBB integrity by the two glial cell types. The latter opens the possibility of manipulating AQP4 levels as a therapeutic option to maintain vascular integrity under different neurodegenerative/neuroinflammatory conditions, in agreement with a recent study supporting the modulation of its subcellular localization for the treatment of brain edemas [46]. A limitation of the current study is that the effect of systemic inflammation on the extent of blood vessel coverage is being studied in each glial cell population separately, thus not allowing a direct correlation of the two cell populations’ mutual dynamics. To decipher the exact extent of the contribution of reactive, vasculature-associated microglia to the retraction of astrocytic endfeet from vessel walls would demand a combined temporal analysis of both astrocytic and microglial populations, ideally including BBB integrity assessment, using 2-photon microscopy the first 4 days after LPS administration. Another limitation of the study is that it is mostly descriptive; therefore, functional validation of AQP4 contribution on the extent of astrocytes and microglia contact to the vasculature following systemic inflammation, through its knock-out, would be required to ensure its key role in the maintenance of BBB integrity. Further study in the above directions is required to fully uncover the interactions of reactive perivascular astrocytes and microglia within the frame of the complex NVU cellular system following systemic inflammation.

## Data Availability

Fiji macros and notebooks for the entire image analysis pipeline are available at the public code hosting website: https://gitlab.pasteur.fr/iah-public/ptrmiad-thomaidou (accessed on 29 March 2023).

## Supplementary Materials

The following supporting information can be downloaded at https://www.mdpi.com/article/10.3390/cells12101418/s1, Videos S1–S3: 3D reconstruction of a microglial cell in control mice (Video S1), 4 days (Video S2) and 8 days (Video S3) following LPS administration; Videos S4–S6: 3D reconstruction of an astrocyte in control mice (Video S4), 4 days (Video S2) and 8 days (Video S3) following LPS administration.
